# Topical ocular application of aggrelyte-2A reduces lens stiffness in mice

**DOI:** 10.3389/fopht.2023.1274825

**Published:** 2023-10-31

**Authors:** Sudipta Panja, Mi-Hyun Nam, Hanmant Gaikwad, Johanna Rankenberg, Ram H. Nagaraj

**Affiliations:** ^1^ Department of Ophthalmology, School of Medicine, Sue Anschutz-Rodgers Eye Center, University of Colorado Anschutz Medical Campus, Aurora, CO, United States; ^2^ Department of Pharmaceutical Sciences, Skaggs School of Pharmacy and Pharmaceutical Sciences, University of Colorado Anschutz Medical Campus, Aurora, CO, United States

**Keywords:** lens, disulfides, acetylation, *N^ε^*-acetyllysine, stiffness, presbyopia

## Abstract

Presbyopia is the progressive loss of the ability of the lens to focus on nearby objects due to its increased stiffness. It occurs in the mid-40s and continues to worsen until the mid-60s. The age-associated increase in protein cross-linking in the lens leads to protein aggregation and water insolubility, especially in the nuclear region, contributing to lens stiffness. This study reports the development of aggrelyte-2A (methyl *S*-acetyl-*N*-(3,3-dimethylbutanoyl) cysteinate, a derivative of our previously reported aggrelyte-2) for reversing the stiffness of aged lenses. Aggrelyte-2A showed minimal toxicity in cultured mouse lens epithelial cells (up to 2000 µM) and human lens epithelial cells (up to 250 µM). Lenses from aged mice (age: 24-25 months) treated with 1 mM aggrelyte-2A for 24 h, and human lenses (age: 47-67 years) treated with 250 µM aggrelyte-2A for 48 h showed 11-14% reductions in stiffness, accompanied by an increase in acetyllysine in lens proteins, and free-thiols in the lens. Topical application of aggrelyte-2A (40 mM, 5 µl twice daily for 4 weeks) on mouse eyes significantly reduced lens stiffness. The topical application showed no toxicity to the lens, cornea, or retina, as revealed by morphological examination, H&E staining, and optical coherence tomography. These data suggest that aggrelyte-2A could be developed as a presbyopia-reversing therapeutic.

## Introduction

Presbyopia is a condition where the eyes gradually lose their ability to focus on nearby objects. It is a natural result of aging and typically begins in the mid-40s (1). This condition occurs in 128 million (32%) and 1.8 billion (25%) people in the US and worldwide, respectively (1–3). The inability to read without corrective eyeglasses reduces productivity, especially in underdeveloped and developing countries (4). Thus, several laboratories and pharmaceutical companies are developing therapeutics to reverse presbyopia (5).

The lens is a protein-rich tissue, with protein concentrations of approximately 400 mg/mL (6, 7). The short-range spatial order of these proteins, predominantly crystallins, accounts for lens transparency. However, crystallins have a limited turnover rate in the lens and this leads them to accumulate post-translational modifications throughout life (8, 9). Such modifications can result in protein cross-linking, aggregation, and water insolubility (9, 10). These changes, which lead to the stiffening of the lens, are more prevalent in the nucleus of the lens, which contains older proteins, compared to the cortex of the lens (11, 12).

The increased lens stiffness or loss of resilience is a major underlying cause of presbyopia (13, 14). The cross-linked, aggregated, and water-insoluble proteins are likely major contributors to such stiffness increase in aging lenses (15, 16). Therefore, it is reasonable to hypothesize that agents that can reverse protein aggregation could reverse presbyopia. Previously, we reported the development of two small molecules, aggrelyte-1 and -2, with properties that increased the protein solubility of water-insoluble proteins and decreased lens stiffness *ex vivo* (17, 18). We ascribed these properties to their ability to acetylate lysine residues and reduce disulfide bonds in lens proteins. However, whether they can reduce lens stiffness *in vivo* has not been tested. Here, we report the development of aggrelyte-2A, a derivative of aggrelyte-2, that shows higher cell permeability than aggrelyte-2, and its effects on lens stiffness *ex vivo* and *in vivo* in mice.

## Materials and methods

### Chemicals and reagents

N,S-diacetyl-L-cysteine methyl ester (aggrelyte-2) (Cat# D95910) was obtained from Astatech (Bristol, PA). L-Cysteine methyl ester (Cat# 410209) was purchased from Sigma-Aldrich (St. Louis, MO). Monoclonal antibodies against Nε-acetyllysine (AcK, Cat# 9681S) and horseradish peroxidase (HRP)-conjugated anti-mouse IgG (Cat# 7076S) were obtained from Cell Signaling Technology (Danvers, MA). All other chemicals used were of at least analytical grade.

### Synthesis of aggrelyte-2A

**Figure 1:**
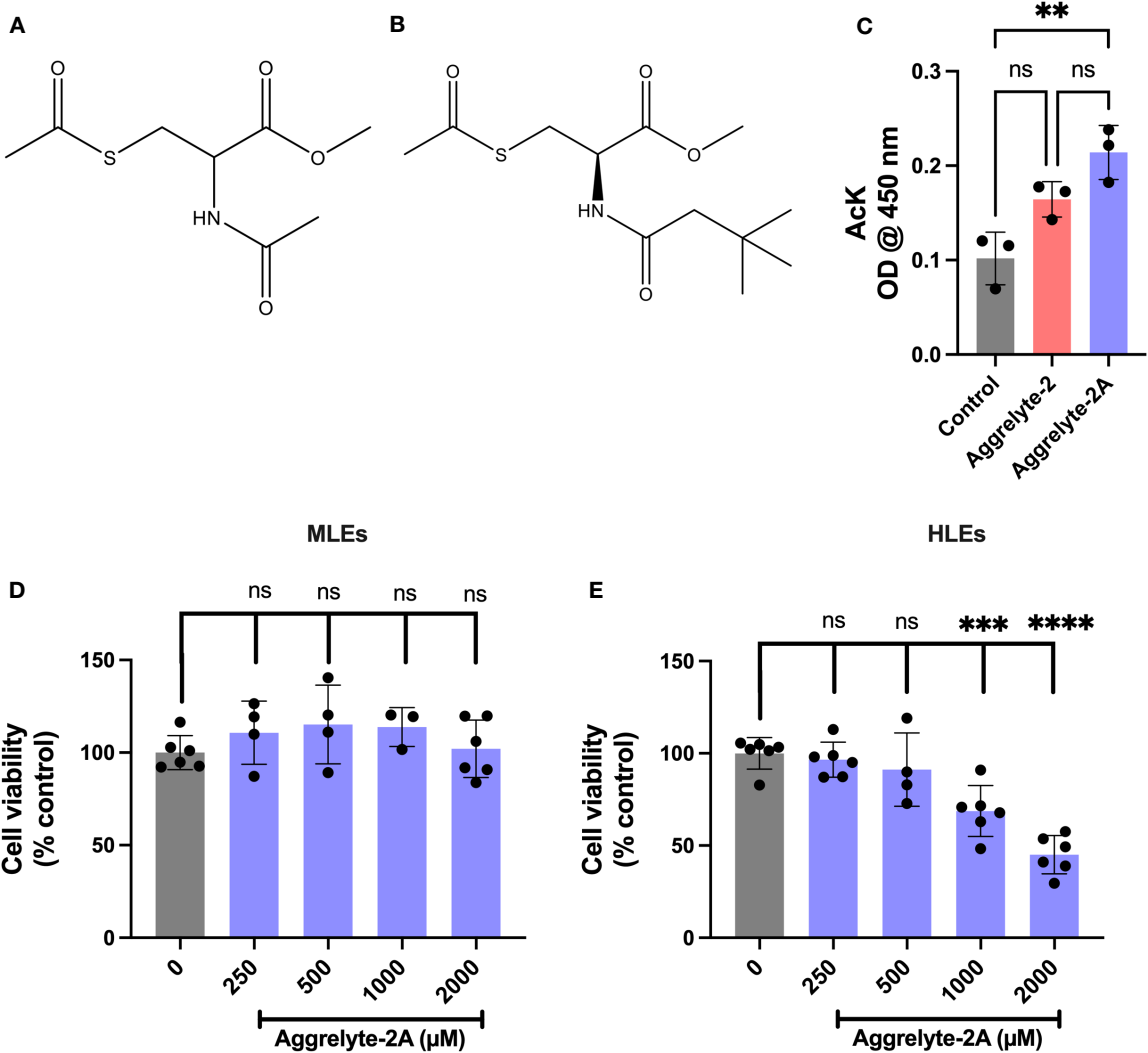
Structure, cell penetration, and cytotoxicity of aggrelyte-2 and -2A. Structure of aggrelyte-2 (N,S-Diacetyl cysteine methyl ester) **(A)** and aggrelyte-2A (methyl *S*-acetyl-*N*-(3,3-dimethylbutanoyl) cysteinate) **(B)**. FHL124 cells were treated in the presence or absence of aggrelytes (250 µM) for 24h. The AcK content in cell lysates was measured by an ELISA **(C)**. Primary mouse lens epithelial cells (isolated from 1-month-old C57BL/6J mice, passages 3-5) were treated with aggrelyte-2A for 24 h **(D)**. Primary human lens epithelial cells (isolated from a 47-year-old non-cataractous lens (passages 3-5) were treated with aggrelyte-2A for 24 h **(E)**. Cell viability was tested by the MTT assay. The bar graphs represent the mean ± S.D. of measurements. **p< 0.01, ***p< 0.001, ****p< 0.0001, ns, not significant.

*N*-acetylation of *L*-cysteine methyl ester (500 mg, 2.91 mmol) was performed by mixing with tert-butylacetyl chloride (364 µl, 2.62 mmol) in *N*,*N*-diisopropylethylamine (456 µl, 2.62 mmol) and stirring overnight at room temperature (RT). The reaction mixture was subjected to prep HPLC on a C18-Safar Biotage 100Å column (solvent A: water + 0.1% TFA and solvent B: 95% acetonitrile in water + 0.1% TFA, 0-7 min: 0-10% B, 7-17 min: 10-100% B, flow rate 15 mL/min). The column eluent was monitored at 215 nm. The product eluted at a R_t_ of 9.7 min was collected and lyophilized. The yield was 76%. S-acetylation of the product was performed by mixing it with acetyl chloride (242 µl, 1.75 mmol) and triethylamine (243 µl, 1.75 mmol) in 20 mL of dichloromethane while stirring at room temperature for 12 h. The solvent was removed under vacuum, and the resulting gummy residue was then purified using prep HPLC (column, solvents solvent gradient and flow rate, as above). The column eluent was monitored at 215 nm, and the product eluting at a R_t_ of 12.9 min was collected and lyophilized. The yield was 89%. The structure of the compound was confirmed by ^1^H-NMR 400 MHz in CDCl_3_c δ 1.03 (s, 9H, CH_3_), 2.10 (s, 2H, CH_2_), 2.34 (s, 3H, CH_3_CO), 3.35 (m, 2H, CH_2_), 3.75 (s, 3H, CH_3_OCO), 4.76 (m, 1H, CH), 6.28 (bs, 1H, CONH). The structure of methyl *S*-acetyl-*N*-(3,3-dimethylbutanoyl) cysteinate (aggrelyte-2A) is shown in **Figure 1**, along with aggrelyte-2.

### Toxicity studies

Mouse lens epithelial cells (MLEs; primary cells from lenses of 1-month-old C57BL/6J mice, passages: 3-5) were incubated with 0-2000 µM aggrelyte for 24 h. Primary human lens epithelial cells (HLEs; isolated from 47-year-old donor lenses, passages: 3-5) were incubated with 0-2000 µM aggrelyte for 48 h, with a change of the media containing freshly dissolved aggrelyte after 24 h. Cell cytotoxicity was measured using the MTT assay.

### Effect of aggrelyte-2A on stiffness in *ex-vivo* cultured mouse lenses

All animal experiments adhered to the ARVO Statement for the Use of Animals in Ophthalmic and Vision Research and were reviewed and approved by the Institutional Animal Care and Use Committee (IACUC) of the University of Colorado, Aurora. The study utilized mice (C57BL/6NTac, 24-25 months old, Taconic Biosciences, Germantown, NY). The lenses were dissected from the eyes by a posterior approach and incubated with or without aggrelyte (1 mM) for 24 h in serum-free and phenol-red-free MEM (297 mOsm) at 37°C. Lens stiffness was measured as previously described (19). Briefly, the lens was placed on a flat platform with the anterior side facing up, and a load was applied from above at 50 mg increments. The displacement of the linear actuator, controlling the decrease in the lens axial diameter, was recorded using MATLAB, with MATLAB software developed by Dr. Adrian Glasser, UK, and plotted against the load applied (19). The displacement at a specific load was used to determine differences in lens stiffness between the untreated or vehicle-treated control and aggrelyte-treated lenses.

### Effect of aggrelyte-2A on the levels of AcK-bearing proteins in mouse lenses

The lenses were homogenized using N_2_-bubbled 50 mM phosphate buffer, pH 7.4 (0.4 mL per lens) in a hand-held motorized homogenizer. The homogenate was centrifuged at 20,000 x g for 20 min at 4°C. The resulting supernatant was collected, and its protein content was measured using BCA Protein Assay Kit using BSA as the standard. The soluble protein was subjected to 12% SDS-PAGE under denaturing conditions and the proteins were electrophoretically transferred to a nitrocellulose membrane and blocked with 5% nonfat dry milk. The membrane was then incubated with an AcK antibody (1:7000 dilution) for 16 h at 4°C, followed by HRP-conjugated goat anti-mouse IgG (1:5000 dilution) for 1 h at RT. The chemiluminescence was detected using the Enhanced Chemiluminescence Detection Kit (Cat# 34096, Thermo Scientific, Waltham, MA). The membrane was stained with Ponceau-S to visualize the proteins and normalize AcK-bearing protein to total protein loaded.

The AcK levels in lens proteins were also measured by a direct ELISA. The ELISA plate wells were coated with 2 µg/50 µl water soluble protein (WS) overnight, washed 3X with PBS with 0.05% Tween-20, and blocked with 300 µl of 5% Non-fat dry milk (NFDM) for 2 h. The wells were incubated for 2 h with 100 µl of 1:2500 diluted AcK monoclonal antibody, followed by washing 3X with PBS with 0.05% Tween-20. The wells were then incubated for 2 h with 100 µl of 1:5000 diluted HRP-conjugated anti-mouse rabbit IgG, followed by incubation with 100 µl of 3,3’,5,5’-tetramethylbenzidine. After adding 50 µl of 2M H_2_SO_4_, the wells were read at 450 nm. Blanks (no primary or secondary antibody) and an internal standard (acetylated BSA) were included.

### Effect of aggrelyte-2A on stiffness in *ex-vivo* cultured human lenses

Human lenses were obtained from the Lions Eye Institute for Transplant & Research, Tampa, FL on ice and immediately utilized for experiments upon delivery. To test the effects of aggrelyte on human lenses (age: 47-67 years), lenses were incubated in the media containing freshly dissolved aggrelyte (250 µM) for 48 h, with freshly dissolved aggrelytes replaced after 24 h. One lens from each donor pair was treated with aggrelyte, while the other was used as the control. After incubation, lens stiffness was measured at 250 mg increments of the load, as above.

### Effect of aggrelyte-2A on the AcK content in human lenses *ex vivo*

Each lens was decapsulated and homogenized in 1.5 mL N_2_-bubbled 50 mM phosphate buffer, pH 7.4 in a hand-held glass homogenizer. The homogenate was centrifuged at 20,000 × g for 20 min at 4°C. The protein concentration in the supernatant was measured using the BCA Protein Assay Kit using BSA as the standard. Western blotting of the solubilized protein was performed as described above to measure protein-AcK levels.

### Effect of topically administered aggrelyte-2A on mouse lens stiffness, AcK levels, and thiol content

Six-month-old C57BL/6J mice (Jackson Laboratories, Barr Harbor, ME; males and females), were acclimatized and assigned to receive topical treatment in their right eye for four weeks. A 40 mM solution of aggrelyte-2A was prepared in a formulation that consisted of benzalkonium chloride (0.001%), NaH_2_PO_4_•H_2_O (0.269%), Na_2_HPO_4_ (0.433%), hyperomellose (0.2%), NaCl (0.5%). Aggrelyte-2A in the formulation (5 µl) was applied to the right eye at 8 h intervals (9 AM and 5 PM) twice daily to unanesthetized animals for four weeks. The left eye received only the formulation (5 µl) or was an untreated control. After four weeks, the animals were euthanized by CO_2_ asphyxiation followed by cervical dislocation. The eyes were enucleated, and the lenses were removed immediately by a posterior approach and placed in fresh PBS. The stiffness of the lenses was measured using a computer-controlled lens squeezer, as described above. The AcK-bearing protein levels were measured by western blotting, as described above. The protein-thiol content was measured using a Thiol Quantification Assay Kit from Abcam (Cat# AB112158), using reduced GSH as the standard, following the manufacturer’s instructions.

### Spectral-domain optical coherence tomography

Mice treated with aggrelyte-2A for 4 weeks were anesthetized using isoflurane. Phenylephrine (2%) and tropicamide (0.2%) drops were applied to the eye to dilate the pupil. Corneal cross-sectional scans (A-Scan/B-scan: 1000 lines, B-scan: 100 scans, Frames/B-scan: 4 frames) were obtained using an AS-OCT (Bioptigen, Durham, NC, USA), and the central corneal thickness was measured using Bioptigen software.

### Electroretinography

ERGs were recorded in mice after a dark adaptation for 2 h. The mice were anesthetized by intraperitoneal injection of ketamine/xylazine. Topical application of tropicamide and phenylephrine dilated pupils. GenTeal gel (Alcon Laboratories, Fort Worth, TX, USA) was used to prevent corneal drying, and the body temperature of the mice was maintained at 37°C. The ERG recordings were obtained using a Celeris rodent ERG system (Diagnosys). To measure scotopic ERG responses, single flashes with intensities of 0.01 cd·s/m^2^ and 0.1 cd·s/m^2^ were employed on the dark-adapted mice. For mesopic responses, a flash intensity of 1.0 cd·s/m^2^ was used. Photopic responses were recorded at intensities of 3 cd·s/m^2^ and 10 cd·s/m^2^. For each flash condition, triplicate responses were collected and averaged.

### Tissue processing and immunohistochemistry

Mice were sacrificed after 4 weeks of topical administration of aggrelyte-2A, the eyes were enucleated and fixed overnight in Davidson’s fixative solution and transferred to 10% Neutral buffered formalin (NBF) for two hours, then treated with 70% ethanol overnight and embedded in paraffin. Five micrometers cross-sections were used for Hematoxylin and Eoisin (H&E), and AcK immunofluorescence staining. The H&E stained slides were digitized using a Nikon Ti-E inverted microscope (Tokyo, Japan) with the 10x objective. Following deparaffinization, the sections were stained with AcK antibody (dilution 1:200) in PBS containing 1% BSA (Millipore Sigma, A1498, St. Louis, MO) and 0.3% Triton X-100 (Acros Organics, New Jersey, NJ) overnight at 4°C. The next day, the sections were washed and then incubated for 2 h at RT with alexa-488 goat anti-mouse secondary antibody (dilution 1:200 Invitrogen, A11011, Waltham, MA). The slides were washed and counterstained with Vectashield (Vector Laboratories, Inc., Burlingame, CA) containing DAPI. Finally, the slides were imaged under a Nikon Eclipse Ti confocal microscope (Nikon, Tokyo, Japan).

### Statistics

All data are expressed as mean ± standard deviation (SD) from at least triplicate samples. Paired and unpaired Student’s t-tests were used to calculate the significance of the difference between samples.

## Results

### Cell-penetrating property of aggrelyte-2A

The structure of aggrelyte-2 and -2A are shown in **Figures 1A, B**. We tested the ability of aggrelytes to penetrate HLEs. FHL124 cells were incubated with aggrelyte-2 and -2A, each at 250 µM concentration for 24 h. The AcK levels in the cell lysates were measured by a direct ELISA. The AcK levels were 1.3-fold higher with aggrelyte-2A treatment than with aggrelyte-2 treatment (**Figure 1C**), suggesting that aggrelyte-2A has better permeability than aggrelyte-2 in HLEs. The addition of the tert-butyl group to aggrelyte-2A made the compound more hydrophobic and may have contributed to its greater cell-penetrating ability than its parent compound aggrelyte-2. This enhanced cell penetrance property was one of our considerations in selecting it for further testing with human lenses *ex vivo* and topical application with mice.

### Toxicity of aggrelyte-2A towards lens epithelial cells

Aggrelyte-2A exhibited no significant toxicity to primary MLEs when tested for 24 h, even at concentrations up to 2000 µM (**Figure 1D**). On the other hand, HLEs were found to be more sensitive compared to MLEs. At 24 h, slight toxicity was observed at 500 µM, with 91% of the cells remaining compared to the untreated controls. Beyond that concentration, both 1000 µM and 2000 µM, showed significant toxicity to cells (**Figure 1E**). At 48 h, with 86% of the cells remaining compared to the untreated controls the treatment with 250 µM did not show statistically significant toxicity (**Figure S2**). However, the toxicity at 500 µM was significant. Based on these results, in all subsequent experiments, we utilized concentrations of 1000 µM for MLEs and 250 µM for HLEs.

### The ability of aggrelyte-2A to reduce lens stiffness *ex vivo*

**Figure 2:**
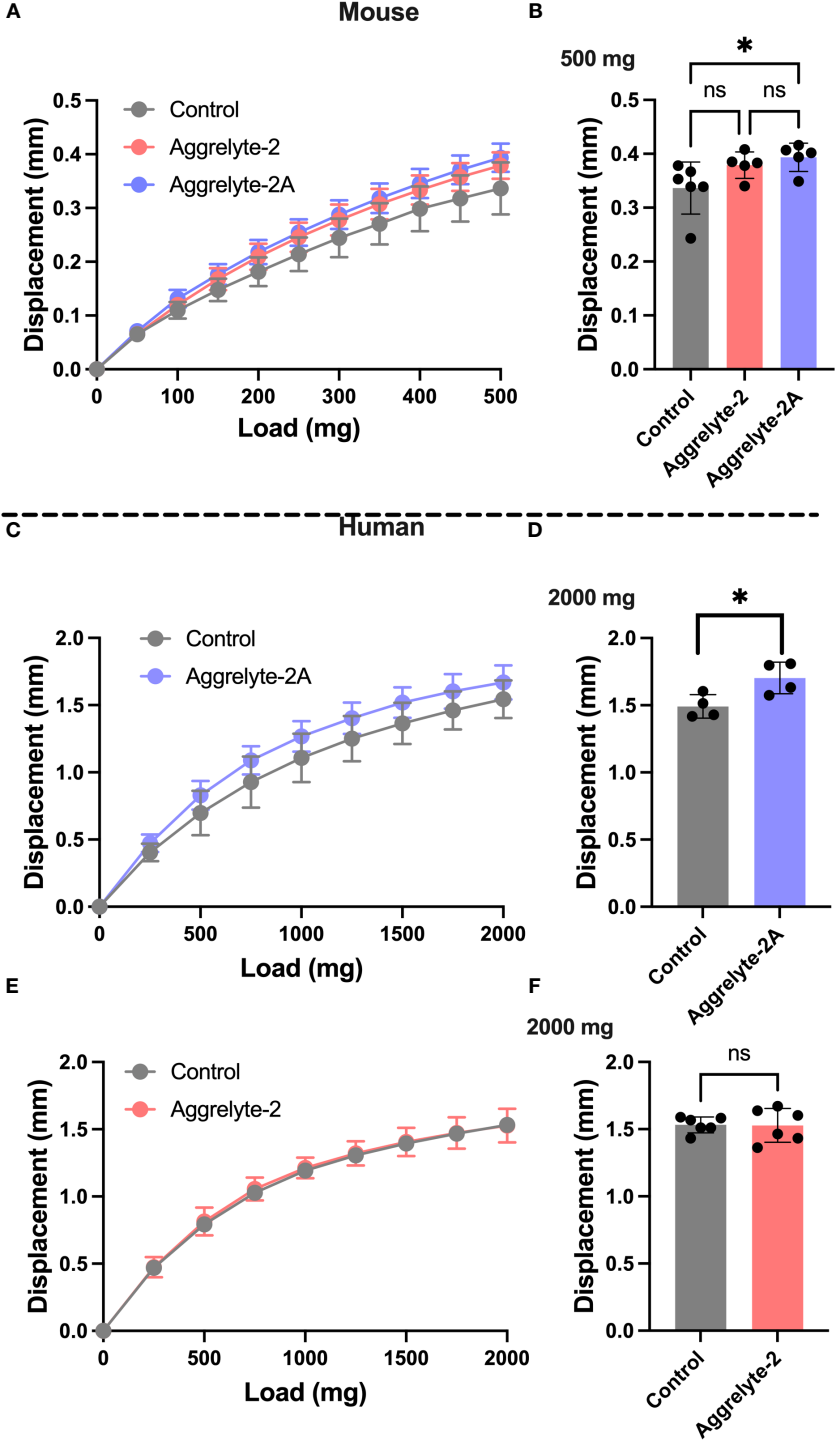
Aggrelyte-2A is more effective than aggrelyte-2 in reducing the stiffness of mouse and human lenses ex vivo. Mouse lenses (from 24-25-month-old C57BL/6NTac mice, n = 5-6) were incubated with or without (control) aggrelyte-2A or aggrelyte-2 (1 mM) for 24 h in serum-free MEM. Freshly obtained human lenses (47-67 years old, n=5) were incubated without (control) or with aggrelyte-2A or 2 (250 µM) for a total of 48 h in serum-free MEM with a change in media containing freshly dissolved aggrelytes every 24 h. Lens stiffness was measured as described in Methods. The displacement in axial lens diameter as a function of change in the load applied is shown in **(A)** (mouse), **(C, E)** (human). The bar graph for mouse lenses shows changes in displacement at a 500 mg load applied **(B)**. Displacements for treated human lenses at a load of 2000 mg are shown on the right; aggrelyte-2A **(D)** and aggrelyte-2 **(F)**. The controls in each case were contralateral lenses incubated without aggrelyte. The bar graphs represent the mean ± S.D. of measurements. *p< 0.05, ns, not significant.

We compared the abilities of aggrelyte-2 and -2A to reduce lens stiffness in *ex vivo* cultured mouse and human lenses. When incubated with lenses of aged mice (age: 24-25 months) at 1000 µM concentration for 24 h, aggrelyte-2 and -2A reduced the stiffness of mouse lenses. At 200 mg load applied, the reduction in stiffness was 15% in the case of aggrelyte-2, and 20% in the case of aggrelyte-2A (p=0.07 compared to controls, **Figure 2A**). In comparison to the control group (**Figure 2B**), aggrelyte-2A significantly reduced lens stiffness at a 500 mg load (p=0.05). This was not observed with aggrelyte-2. Despite this improved effect, the result for aggrelyte-2A was not statistically different from aggrelyte-2. The human lenses (age: 47-67 years) treated with 250 µM aggrelyte-2A for 48 h exhibited a significant reduction in stiffness of 12% at a load of 1000 mg (p=0.20) and by 14% at 2000 mg load (p=0.05, **Figures 2C, D**), while no reduction in stiffness was observed in the lenses treated with aggrelyte-2 at these loads (**Figures 2E, F**). Together, these results suggested that at equal concentrations, aggrelyte-2A has a greater ability to reduce stiffness in mouse and human cultured lenses relative to aggrelyte-2.

### AcK and thiol levels in aggrelyte-2A treated lenses

**Figure 3:**
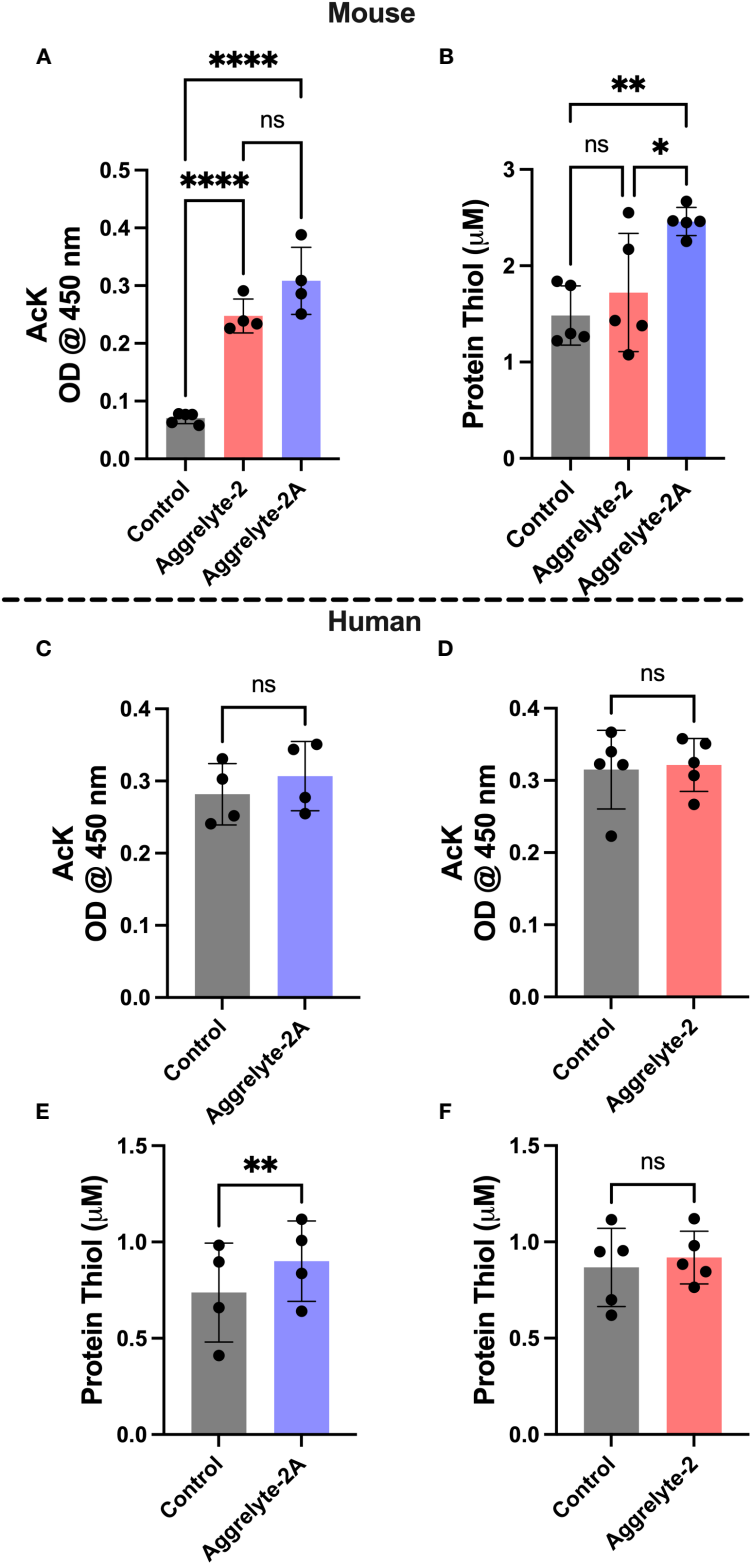
Treatment with aggrelyte-2A increases the content of AcK-bearing proteins and free protein thiol levels in mouse and human lenses. Lenses were incubated as described in **Figure 2**. The AcK-bearing proteins in water-soluble proteins were measured by a direct ELISA. Aggrelyte-2A increased the acetylation of proteins in mouse **(A)** and human **(C)** lens proteins greater than aggrelyte-2 **(A, D)**. The protein-thiol content in the solubilized protein was measured using a Thiol Quantification Assay Kit as described in the Methods. Aggrelyte-2A treated mouse **(B)** and human **(E, F)** lens protein contained higher levels of free protein thiol content compared to aggrelyte-2. The bar graphs represent the mean ± S.D. of n = 4-5 measurements. *p< 0.05, **p< 0.01, ****p<0.0001, ns, not significant.

The aggrelyte -treated mouse lenses showed significantly higher AcK levels (p<0.0001), as determined by a direct ELISA (**Figure 3A**). Aggrelyte-2A produced approximately 1.2-fold higher levels of AcK than aggrelyte-2. In agreement with the ELISA results, western blot analysis also showed a significant 5.8-fold and 8.0-fold increase in the AcK levels upon treatment with aggrelyte-2 (p<0.05) and aggrelyte-2A (p<0.01), respectively (**Figures S3A, B**). This further suggests that aggrelyte-2A has greater permeability into mouse lenses than aggrelyte-2. There was a modest increase in the AcK levels in human lenses treated with aggrelyte-2A (p= 0.09) compared to untreated lenses (**Figure 3C**). However, no such difference in the AcK levels was observed between the lenses treated with aggrelyte-2 and the untreated controls (p= 0.56; **Figure 3D**). The western blot results also indicated a marginal increase in AcK level (~11%) relative to controls after being treated with aggrelyte-2A (**Figures S3C, D**). However, there was no change in AcK content before and after treatment with aggrelyte-2 (**Figures S3E, F**). The thiol content increased significantly (p<0.01) in mouse lenses treated with aggrelyte-2A (**Figure 3B**); the increase was 1.7-fold and 1.4-fold when compared to controls and aggrelyte-2 treated lenses, respectively. Aggrelyte-2A treated human lenses showed a significant 1.2-fold increase (p<0.01) in the thiol content upon treatment with aggrelyte-2A, but not with aggrelyte-2 (**Figures 3E, F**). These results suggested that aggrelyte-2A produces higher AcK levels and reduces more disulfide bonds than aggrelyte-2 in human and mouse lenses. Although aggrelyte-2A treatment led to increased levels of AcK and free thiols in mouse lenses, the reduction in stiffness was similar to that seen with aggrelyte-2. It is unclear why there is a discrepancy between the levels of AcK and free thiols and the reduction in stiffness.

### Topical administration of aggrelyte-2A to mouse eyes

**Figure 4:**
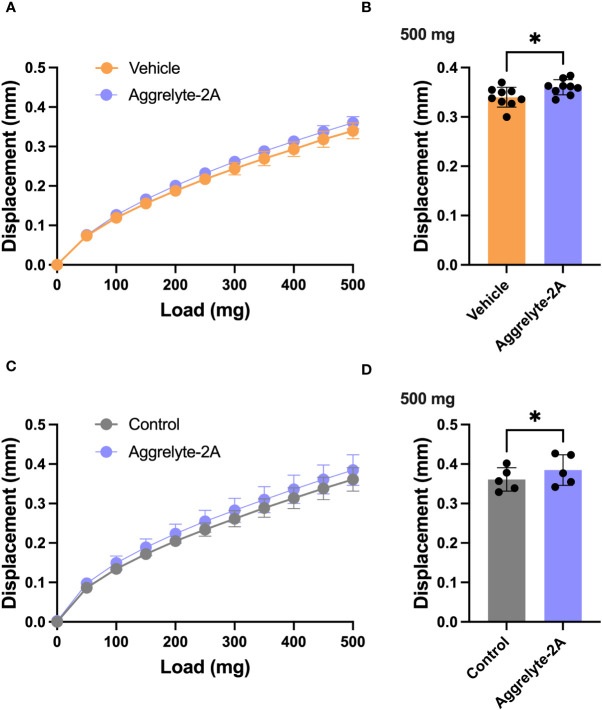
Topical administration of aggrelyte-2A reduces the stiffness of mouse lenses. C57BL/6J mice, 6 months old, were acclimated and assigned to receive topical treatment in their right eyes for 4 weeks with a 40 mM aggrelyte-2A ophthalmic formulation, while the left eyes received a formulation vehicle or were untreated controls as described in Methods. Lenses were removed immediately after sacrificing animals and placed in PBS. Lens stiffness was measured as described in Methods. The displacement in lens diameter as a function of the load applied is shown in **(A, C)** The bar graphs show changes in displacement at a 500 mg load in the vehicle and aggrelyte-2A treated **(A, B)** and control and aggrelyte-2A treated **(C, D)** treated lenses. The bar graphs represent the mean ± S.D. of measurements of n= 5-9 lenses. *p< 0.05.

**Figure 5:**
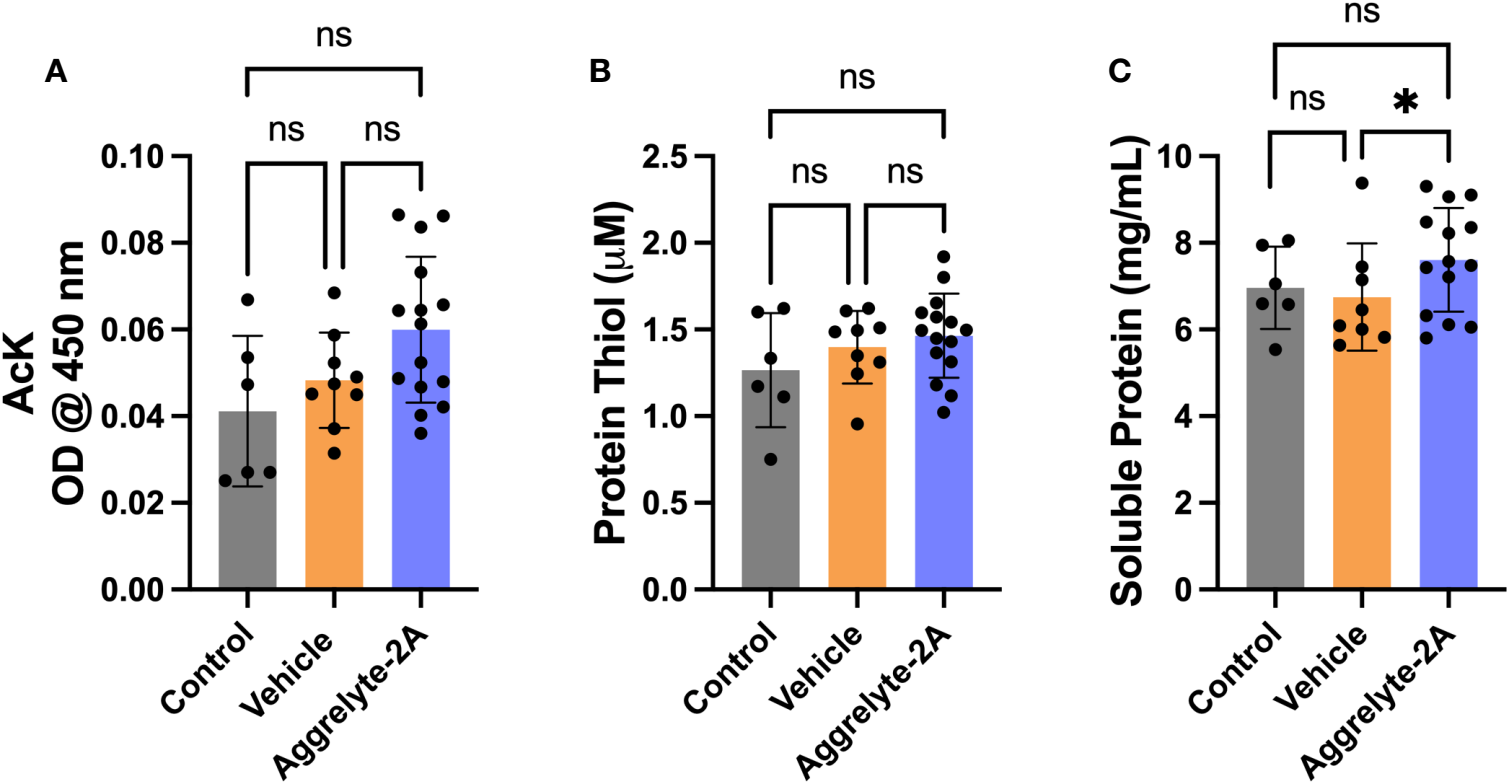
Topical administration of aggrelyte-2A increases AcK levels and free protein thiol levels in mouse lenses. The AcK-bearing protein levels in the water-soluble proteins of aggrelyte-2A treated mouse lenses were measured by a direct ELISA. Aggrelyte-2A treatment resulted in modest increases in the protein AcK levels **(A)**. The thiol content **(B)** and soluble protein levels **(C)** were measured as described in Methods. The bar graphs represent the mean ± SD of measurements. Statistical significance is indicated by *p<0.05, ns, not significant.

Since aggrelyte-2A was better than aggrelyte-2 in reducing stiffness in cultured mouse lenses, we tested only aggrelyte-2A for the topical application studies. Topical application of aggrelyte-2A to the eyes of 6-month-old mice for 4 weeks reduced lens stiffness by 7.3% and 8.7% at 250 mg load and 6.2% and 6.5% at 500 mg load relative to the contralateral vehicle or control lenses (**Figures 4A-D**). Remarkably, when compared to vehicle-treated or control lenses, the effect of aggrelyte-2A was statistically different (p<0.05). The AcK levels, measured by direct ELISA, although not significantly different, were 1.2 and 1.5-fold higher in the aggrelyte-2A treated lenses than in vehicle-treated or control lenses (**Figure 5A**). The thiol content was 4.7% and 15.7% higher in aggrelyte-2A treated lenses than in the vehicle alone treated and control lenses (**Figure 5B**). The soluble protein content in aggrelyte-2A treated lenses was 9.0% and 12.9% higher than control and vehicle (p<0.05) treated lenses (**Figure 5C**). These results suggested that topically administered aggrelyte-2A reduces lens stiffness through lysine acetylation and disulfide reduction.

### AcK modification in mouse eyes after topical application of aggrelyte-2A

**Figure 6:**
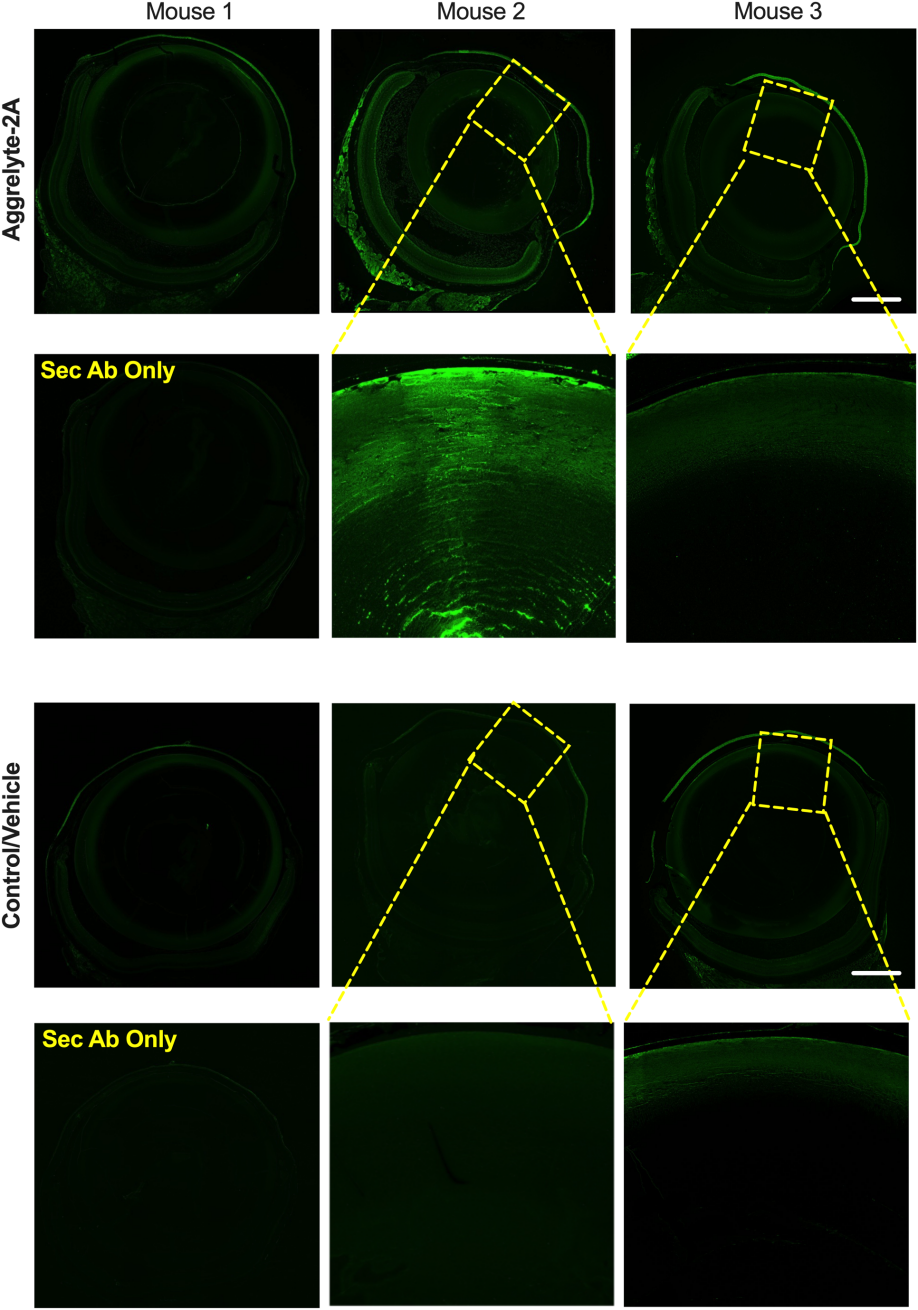
Effect of topical administration of aggrelyte-2A on AcK-modified proteins in the mouse eye. Immunohistochemical analysis of transverse sections of three mouse eyes treated topically with aggrelyte-2A as described in Methods revealed increased immunoreactivity for AcK (green) in comparison to untreated (controls) or vehicle-alone treated eyes. The omission of the primary antibody resulted in the absence of AcK immunoreactivity. The scale bar corresponds to 500 μm.

We performed AcK immunofluorescence analysis on cross-sections of mouse eyes. In contrast to control and vehicle-alone treated eyes that showed minimal or no AcK-specific fluorescence, we observed varying levels of fluorescence in aggrelyte-2A treated lenses (**Figure 6**). At higher magnifications, we observed AcK immunofluorescence in the nucleus, but the AcK levels were higher in the cortex than in the nucleus (middle image of mouse 2). We also observed a noticeable increase in the AcK fluorescence signal in the cornea and retina in aggrelyte-2A-treated mouse eyes.

### Effect of topical application of aggrelyte-2A on morphology and retinal function in mouse eyes

Based on the OCT images taken after topical applications of aggrelyte-2A in mice, there were no indications of corneal edema. Additionally, the thickness of the cornea remained unchanged (S4 A-C). The lenses remained clear (**Figures S4D, E**). Furthermore, H&E staining revealed no morphological changes in lenses (**Figures S4F, G**). No morphological abnormalities were observed in the cornea and retina (**Figures S4F-I**). Additionally, ERG analyses showed no difference in scotopic, mesopic, and photopic retinal responses between the eyes treated with aggeryte-2A and those treated with the vehicle (**Figure S5**). Overall, these results indicated that the topical application of aggrelyte-2A at a concentration of 40 mM for 4 weeks in mice did not have any detectable adverse effects on the eye.

## Discussion

The aim of this research was to enhance the cell permeability of aggrelyte-2 and investigate if aggrelyte-2A, when applied topically, could decrease lens stiffness in mice. The addition of a tert-butyl group to aggrelyte-2 (aggrelyte-2A) resulted in a 30% improvement in cell permeability in comparison to aggrelyte-2. This was determined by analyzing the AcK levels in FHL124 cells.

We tested the ability of aggrelyte-2A to reduce stiffness in cultured mouse and human lenses. Aggrelyte-2A was able to reduce stiffness in aged mouse lenses by 17.0% (measured at 500 mg load) and human lenses by 14.2% (measured at 2000 mg load) at 1000 µM and 250 µM, respectively. In comparison, aggrelyte-2 reduced mouse lens stiffness by 12.6% and was ineffective in reducing human lens stiffness at similar concentrations and loads applied. Aggrelyte-2A has shown promising potential for reducing lens stiffness even at a lower concentration of 250 µM, which is 4 times lower than that of aggrelyte-2. This makes it a viable candidate for further testing in humans.

In mice, topical application of aggrelyte-2A significantly reduced lens stiffness by 7.3% and 8.7% at 250 mg load and 6.2% and 6.5% at 500 mg load compared to the contralateral vehicle and control lenses, respectively. This is likely due to lysine acetylation and disulfide reduction, as indicated by the increase in AcK levels in the lens and free thiol groups. There were no harmful effects on the cornea and retina, with no changes observed in retinal functions measured by ERG and corneal thickness measured by OCT. We did, however, notice higher AcK content in the corneal epithelial cells, but it remains to be seen if this impacts their functions. If the cells possess robust lysine deacetylase activities, the modifications could be reversed, and any adverse effects of aggrelyte-2A may be temporary and reversible. Moreover, the regular replacement of corneal epithelial cells may reduce any negative consequences. Further investigation is necessary to determine the exact impact of AcK levels on epithelial cell functions.

The stiffness reduction of mouse lenses from the topical application of aggrelyte-2A was not as significant as the reduction achieved by lipoic acid choline ester (LACE) (20). However, we used a concentration of aggrelyte-2A that was five times less than the concentration of LACE. Additionally, we utilized comparatively younger mice for the topical administration. Determining the ideal amount of stiffness reduction in lenses to reverse presbyopia in humans is a challenging task. The lack of animal models for human presbyopia is an obstacle. Nevertheless, in mice, we applied aggrelyte-2A topically and achieved a significant decrease in lens stiffness. It is uncertain whether reducing the stiffness of presbyopic lenses in humans to a similar extent would be enough to reverse presbyopia, and this would require a potential clinical trial to determine. It is important to consider that a small decrease in stiffness could help to partially or completely reverse presbyopia, while minimizing the risk of harming the structure or function of the lens.

The decrease in lens stiffness caused by the LACE was thought to be due to its ability to decrease disulfide bonds in aggregated lens proteins. Despite promising results in animal testing (20), and Phase I clinical trial (21, 22), LACE (UNR844), did not meet expected standards in a Phase 2b clinical trial. Consequently, Novartis opted to discontinue its development in 2022. It is difficult to determine if the reduction of disulfide bond alone, which is a characteristic feature of LACE, can effectively prevent or reverse presbyopia. This is because there are several other concurrent modifications occurring that could result in protein aggregation in the lens. Aggrelyte-2A, on the other hand, not only reduces disulfides in proteins but also acetylates lysine residues, which is unlikely to be undone in the metabolically inactive lens core. As a result, we anticipate that the lens stiffness reduction achieved by aggrelyte-2A will be superior and longer-lasting. We applied a 40 mM solution of aggrelyte-2A twice for 4 weeks to achieve a significant reduction in lens stiffness in mice. Whether such repetitive applications and concentrations are needed to achieve a similar reduction in stiffness in presbyopic human lenses will need to be determined.

Presbyopia can now be treated with FDA-approved Pilocarpine Ophthalmic Solution (23). Other miotic drugs, similar to pilocarpine, are being tested in clinical trials. These treatments enhance near vision by reducing pupil size, but they must be used frequently for long-lasting effects. Conversely, aggrelyte-2A treatment lowers lens stiffness through protein acetylation and disulfide reduction. This means that the effects of agerelyte-2A are anticipated to last longer than FDA-approved medication and medications currently being developed for presbyopia. There is hope that applying aggrelyte-2A topically for a few weeks or months may even result in the permanent reversal of presbyopia.

In conclusion, we have made improvements to aggrelyte-2 and developed aggrelyte-2A which can reverse stiffening in aged mouse and human lenses. Future studies, which may involve aged non-human primates or other animals that mimic human presbyopic lenses, will determine whether aggrelyte-2A can be advanced to a human clinical trial.

## Data availability statement

The original contributions presented in the study are included in the article/**Supplementary Material**. Further inquiries can be directed to the corresponding author.

## Ethics statement

The requirement of ethical approval was waived by University of Colorado Anschutz Medical Campus for the studies on humans because the study used lens tissue from cadaver donors. The studies were conducted in accordance with the local legislation and institutional requirements. Written informed consent for participation was not required from the participants or the participants’ legal guardians/next of kin in accordance with the national legislation and institutional requirements. The animal study was approved by University of Colorado Anschutz Campus IACUC. The study was conducted in accordance with the local legislation and institutional requirements.

## Author contributions

SP: Writing – original draft, Writing – review & editing, Data curation, Formal Analysis, Investigation, Methodology, Validation. M-HN: Investigation, Methodology, Validation, Writing – original draft, Writing – review & editing, Data curation, Formal Analysis. HG: Data curation, Formal Analysis, Investigation, Methodology, Writing – review & editing. JR: Data curation, Formal Analysis, Investigation, Methodology, Writing – review & editing, Validation, Writing – original draft. RN: Data curation, Formal Analysis, Writing – original draft, Writing – review & editing, Conceptualization, Funding acquisition, Supervision.
