# Supplementary material for: Topical ocular application of aggrelyte-2A reduces lens stiffness in mice
Source: Front Ophthalmol (Lausanne). 2023 Oct 31;3:1274825. doi: 10.3389/fopht.2023.1274825 (PMC11182177; doi:10.3389/fopht.2023.1274825)
Supplement: Supplementary file 1 [file DataSheet_1.pdf]

Supplementary Material

Topical Ocular Application of Aggrelyte-2A Reduces Lens Stiffness in Mice

Sudipta Panja[1], Mi-Hyun Nam[1], Hanmant Gaikwad[2], Johanna Rankenberg[1] and Ram H. Nagaraj[1,2,*]

***Correspondence:** ram.nagaraj@cuanschutz.edu

Supplementary Figures

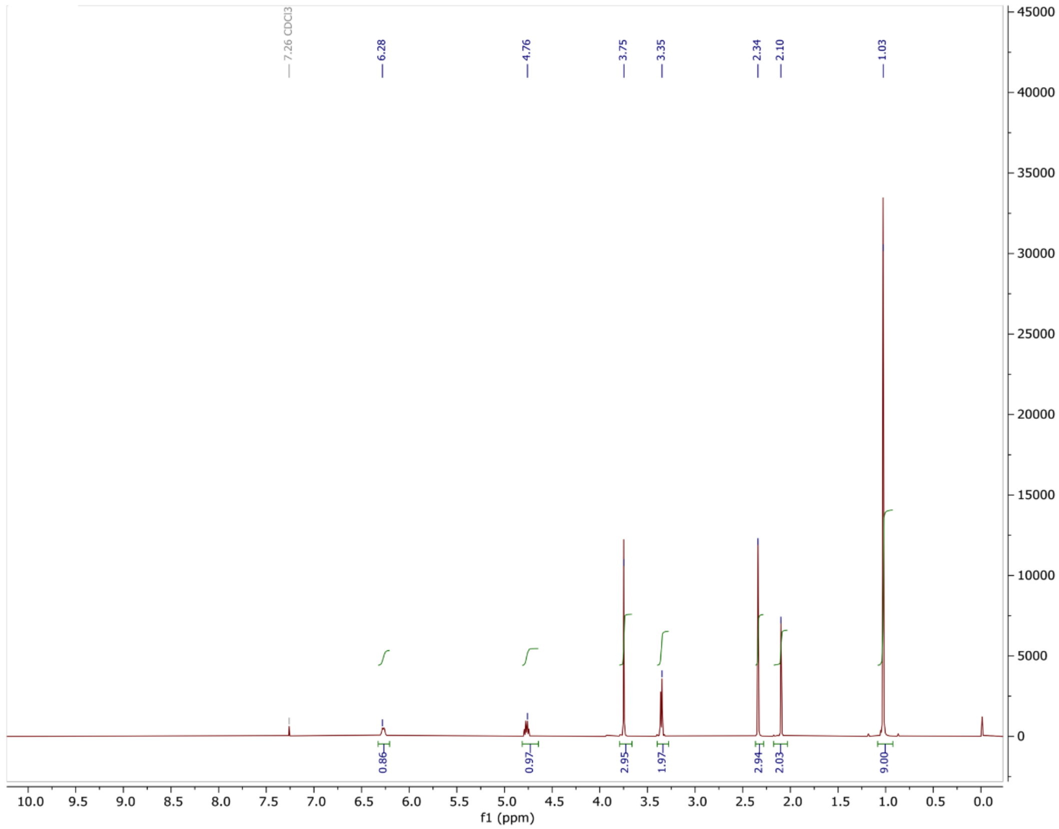

Figure S1. ^{1}H-NMR spectra (400 MHz, $CDCl_3$) of synthesized aggrelyte-2A. δ 1.03 (s, 9H, CH_3), 2.10 (s, 2H, CH_2), 2.34 (s, 3H, CH_3CO), 3.35 (m, 2H, CH_2), 3.75 (s, 3H, CH_3OCO), 4.76 (m, 1H, CH), 6.28 (bs, 1H, CONH).

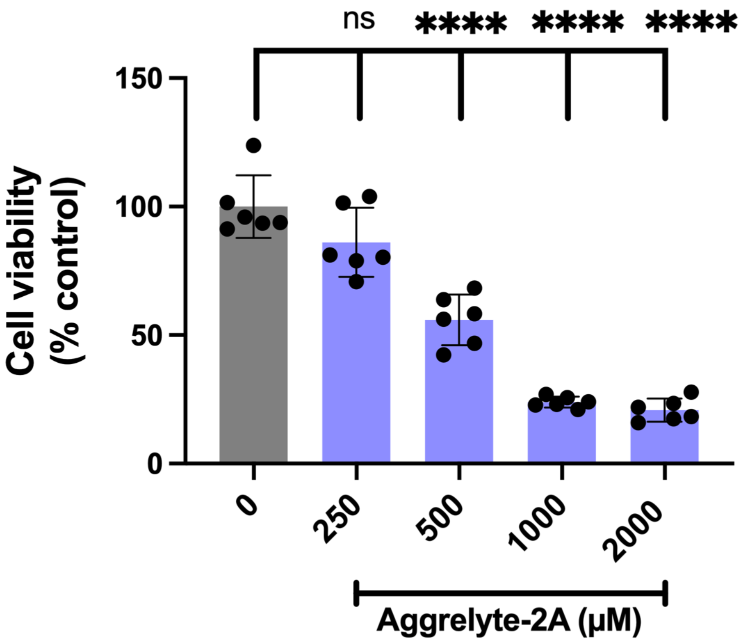

Figure S2. Cytotoxicity of aggrelyte-2A to HLEs. Primary human lens epithelial cells (isolated from a 47-year-old noncataractous lens, passages 3-5) were treated with aggrelyte-2A for 48 h, with a change in media containing freshly dissolved aggrelytes every 24 h. Cell viability was tested by the MTT assay. ****p< 0.0001, ns = not significant.

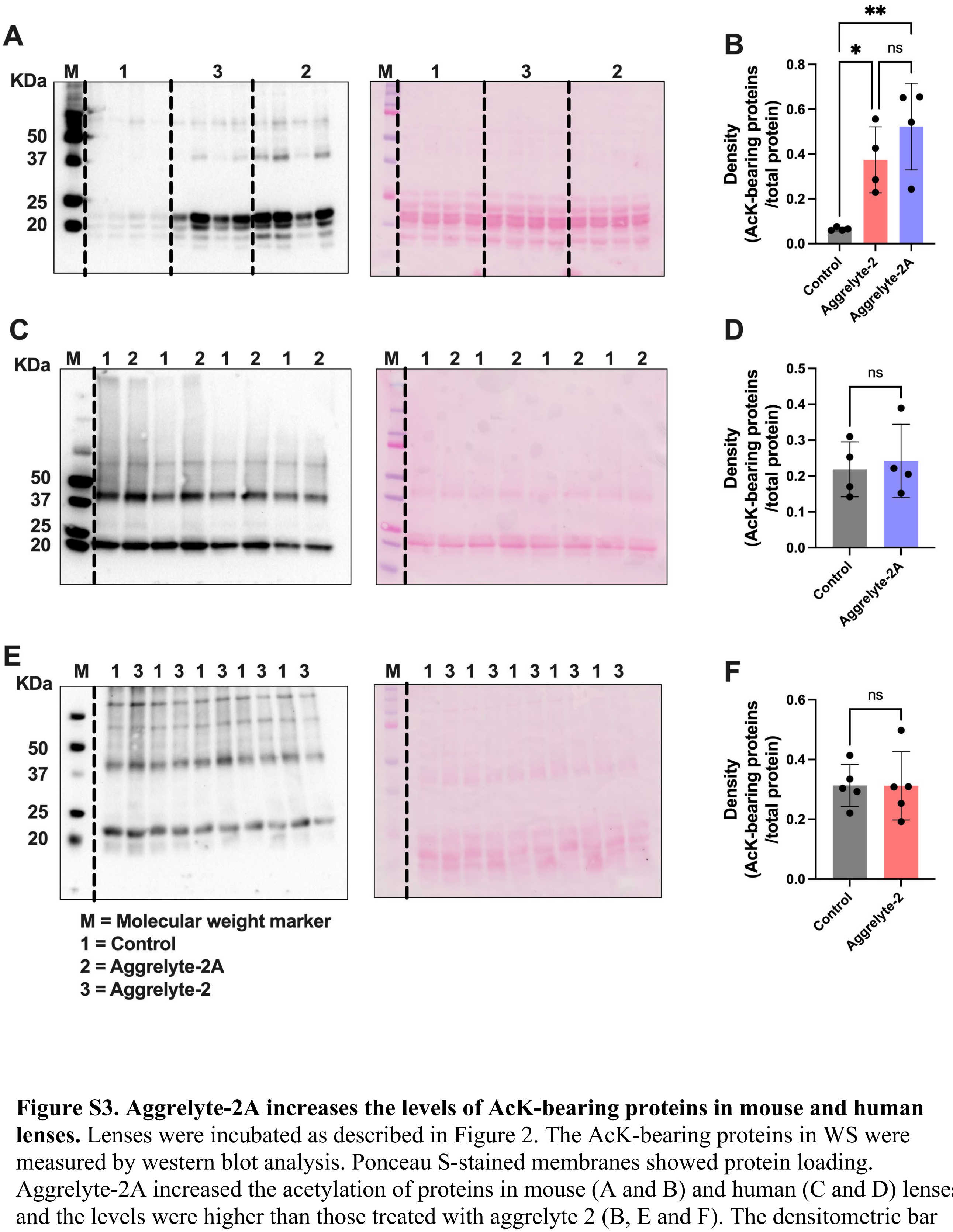

Figure S3. Aggrelyte-2A increases the levels of AcK-bearing proteins in mouse and human lenses. Lenses were incubated as described in Figure 2. The AcK-bearing proteins in WS were measured by western blot analysis. Ponceau S-stained membranes showed protein loading. Aggrelyte-2A increased the acetylation of proteins in mouse (A and B) and human (C and D) lenses and the levels were higher than those treated with aggrelyte 2 (B, E and F). The densitometric bar graphs represent the mean ± S.D. of n = 4 measurements. M =molecular weight markers. *$p < 0.05$, **$p < 0.01$, ns = not significant.

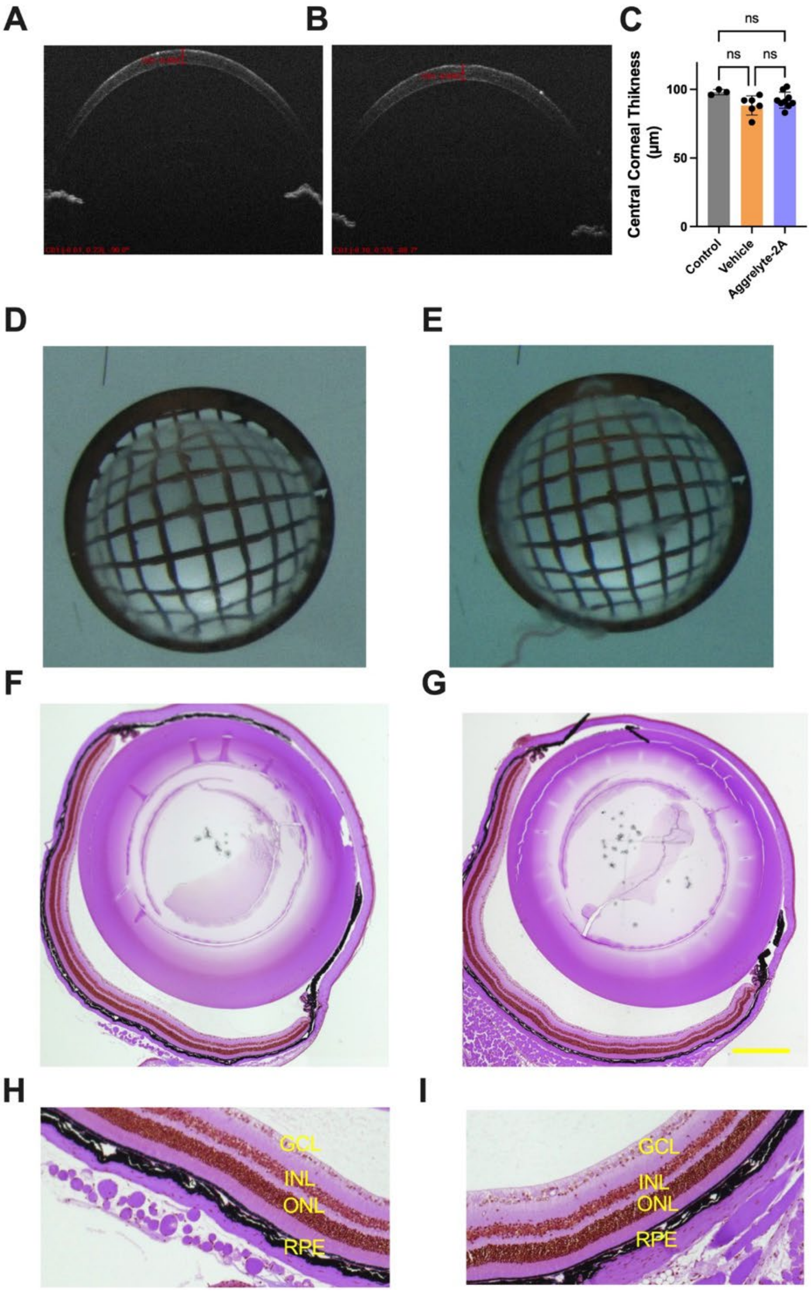

Figure S4. The effect of topical application of aggrelyte-2A on the morphology of the mouse eye. Optical coherence tomography (OCT) images show the cornea of the vehicle **(A)** and aggrelyte-2A **(B)** treated representative mouse eyes. A comparison of the central corneal thickness between aggrelyte-2A treatment, untreated control and vehicle-treated eyes is shown in **(C)**. No change in transparency was observed between the vehicle **(D)** and aggrelyte-2A **(E)** treated lenses. H&E-staining of vehicle **(F and H)** and aggrelyte-2A **(G and I)** showed no changes in the lens morphology **(F and G)** or the retinal architecture **(H and I)**. GCL = ganglion cell layer, INL = inner nuclear layer, ONL = outer nuclear layer, and RPE = retinal pigment epithelium. ns=not significant. The scale bar represents 500 µm.

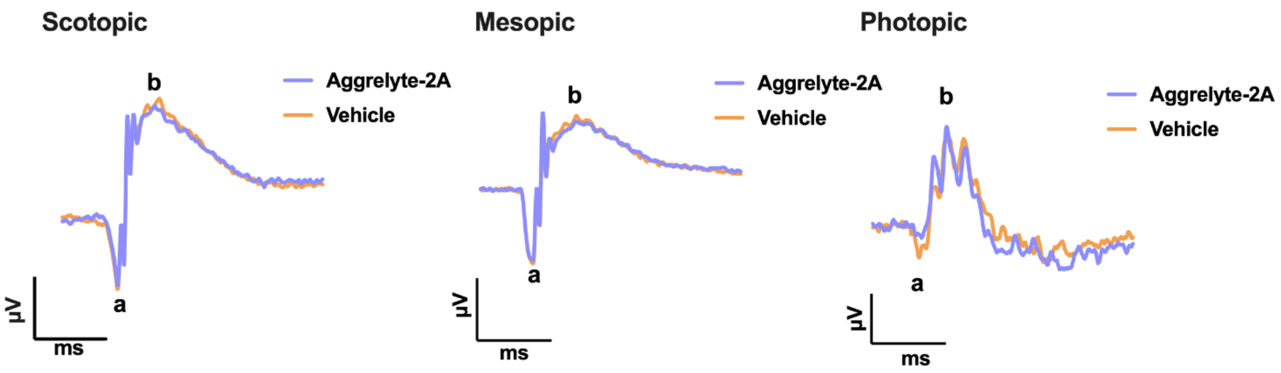

Figure S5. Topical administration of aggrelyte-2A does not change the retinal ERG response. Scotopic, mesopic and photopic electroretinograms (ERGs) were recorded at different light intensities strengths (0.1, 1 and 10 cd·s/m^2) in the vehicle and aggrelyte-2A treated mouse eyes.
